# Supplementary material for: Auxin-regulated chromatin switch directs acquisition of flower primordium founder fate
Source: eLife. 2015 Oct 13;4:e09269. doi: 10.7554/eLife.09269 (PMC4600763; doi:10.7554/eLife.09269)
Supplement: Supplementary file 1. — DOI: http://dx.doi.org/10.7554/eLife.09269.024 [file elife-09269-supp1.docx]

**Supplementary file 1: Primers used in this study**

| **Primer Name** | | **Sequence (5’🡪3’)** | | | |
| --- | --- | --- | --- | --- | --- |
| **Cloning primers**  **Transgenic plants** | | |  | | |
| gMP-F1 | caccgctgctcccaacaatcctac | | | | |
| gMP-R1 | ACAGAGAGATTTTTCAATGTTC | | | | |
| gMP-F2 | agagccccacaagagagaca | | | | |
| gMP-R2 | TTGGCGCGCCactagtTGAAACAGAAGTCTTAAGATCG | | | | |
| gMP-F3 | atgtaacaatataaaaatgatc | | | | |
| gMP-R3 | TTGGCGCGCCatttggctcaccatcacagt | | | | |
| gHDA19-F1 | CACCcctagcttccttgttaacacaccc | | | | |
| gHDA19-R1 | cgACTAGTTGTTTTAGGAGGAAACGCCTGC | | | | |
| gHDA19-F2 | cgACTAGTgactcggagcttctaatttcttgc | | | | |
| gHDA19-R2 | GGCGCGCCttcattgctgcgtttagcac | | | | |
| MPN-F | CACCatgatggcttcattgtcttg | | | | |
| ARF5_fwd | caccATGATGGCTTCATTGTCTTGTGTTGAAG | | | | |
| ARF5∆III/IV_rev | TTAGGTTCGGACGCGGGGTG | | | | |
| **Complex targeting** | | | | |  |
| BSH-F | ccGAGCTC ATGAAGGGTT TAGTGTCTAC | | | | |
| BSH-R | ttGGCGCGCCTCACCTTGCATGTCTCTCTTCC | | | | |
| MPNm2-F | tttcgccatatctacgcagggcaaccaaagaga | | | | |
| MPNm2-R | TCTCTTTGGTTGCCCTGCGTAGATATGGCGAAA | | | | |
| MPNm1-F | gatagtatgcacatcgaagttcttgctgctgct | | | | |
| MPNm1-R | AGCAGCAGCAAGAACTTCGATGTGCATACTATC | | | | |
| BDL-R1 | gaAGATCTctaAACAGGGTTGTTTCTTTGTCTATCC | | | | |
| AXR3-F1 | ATTTGCGGCCGCAATGATGGGCAGTGTCGAGCT | | | | |
| AXR3-R1 | cgGGATCCtcaAGCTCTGCTCTTGCACTTCT | | | | |
| PI-F | CACCATGGGTAGAGGAAAGATCGA | | | | |
| PI-R | TCAATCGATGACCAAAGACAT | | | | |
| **Yeast-two-hybrid, yeast- three-hybrid and BiFC** | | | |  | |
| MP-F1 | CACCGAATTCatgatggcttcattgtcttg | | | | |
| MP-R1 | CCGAGCTCTTATGAAACAGAAGTCTTAA | | | | |
| MP-F2 | CACCatgggaatcagcgatttggatccg | | | | |
| MP-R2 | CCGAGCTCttaGCTTGAAGATGTACCAGTGCC | | | | |
| MP-F3 | CACCGAATTCaatgttgattttgatgattgT | | | | |
| BDL-F1 | ATTTGCGGCCGCAATGCGTGGTGTGTCAGAATTGG | | | | |
| BDL-R1 | gaAGATCTctaAACAGGGTTGTTTCTTTGTCTATCC | | | | |
| AXR3-F1 | ATTTGCGGCCGCAATGATGGGCAGTGTCGAGCT | | | | |
| AXR3-R1 | cgGGATCCtcaAGCTCTGCTCTTGCACTTCT | | | | |
| PI-F | CACCATGGGTAGAGGAAAGATCGA | | | | |
| PI-R | TCAATCGATGACCAAAGACAT | | | | |
| **qRT-PCR** |  | | | | |
| FILRT-F | ACCATCTTTCATGGATCTTCA | | | | |
| FILRT-R | AATCGGTTATATGCGGATGG | | | | |
| TMO3RT-F | CCGAGGAGTGAGACAGCGTCC | | | | |
| TMO3RT-R | GCTTCTTCCGCCGTGTTGTAAGTACC | | | | |
| LFYRT-F | ACGCCGTCATTTGCTACTCT | | | | |
| LFYRT-R | CGCATCAGTCTGGTCTTGTT | | | | |
| ANTRT-F | TGATGGTTCTCTTTGCTTAATGG | | | | |
| ANTRT-R | GGCTTCTTTGTGACTTGTGTTG | | | | |
| EIF4A-1-F | aaactcaatgaagtacttgagggaca | | | | |
| EIF4A-1-R | tctcaaaaccataagcataaataccc | | | | |
| **In situ hybridization** |  | | | | |
| TMO3-F | cgACTAGTATGGAAGCGGAGAAGAAAATGG | | | | |
| TMO3-R | acatGCATGCTTAAACAGCTAAAAGAGGATCC | | | | |
| FIL-F | ATGTCTATGTCGTCTATGTCC | | | | |
| FIL-R | TTAATAAGGAGTCACACCAACG | | | | |
| **ChIP** |  | | | | |
|  |  | | | | |
| FILChIP-Fa | GATAGAAAATAGAACTGGAGAAACCCT | | | | |
| FILChIP-Ra | GTGAATAAAGCATTTACTTATTCTGTGC | | | | |
| FILChIP/FAIRE-Fb | tcttcctccattgggaactg | | | | |
| FILChIP/FAIRE-Rb | ggttgcacgtgatgacacat | | | | |
| FILChIP-Fc | GAAAAAGGTCCATCTTTAAGCG | | | | |
| FILChIP-Rc | agaaattcggttgaccatgc | | | | |
| FILChIP-Fd | ttgtcttattaaacaccggcata | | | | |
| FILChIP-Rd | Agcggtagtgaaccagcaac | | | | |
| FILChIP-Fe | GACCTAGAGGCTACAATTAGAGCAT | | | | |
| FILChIP-Re | GGTGAAAGGAGTGAGAAGGGAAA | | | | |
| TMO3ChIP-Fa | CTTTTATTCTCCTTCTATACATGCGTAC | | | | |
| TMO3ChIP-Ra | CCTAGTTTCCTTCCTACAATGGCA | | | | |
| TMO3ChIP-Fb | ATGGCAGAAGAGACAAGACTCACAA | | | | |
| TMO3ChIP-Rb | CTCTTTCTTGTTTACAGTATGTTTGGTTAG | | | | |
| TMO3ChIP-Fc | tcttgcgatccagtgaaaaa | | | | |
| TMO3ChIP-Rc | catcagttcttgagacaacgataa | | | | |
| TMO3ChIP-Fd | cgcacggaggttatgagatt | | | | |
| TMO3ChIP-Rd | tcgtaattggagattcaggtca | | | | |
| TMO3ChIP-Fe | cgcacggaggttatgagatt | | | | |
| TMO3ChIP-Re | tcgtaattggagattcaggtca | | | | |
| TMO3ChIP-Ff | TCTCTCTCGGCTCTCGCAGTGTT | | | | |
| TMO3ChIP-Rf | GTGGAAAAAGTTTTCTTAAAGGACGC | | | | |
| LFYChIP-Fa | GCTGAATTAAGTCACTTTTGAGTTG | | | | |
| LFYChIP-Ra | TGAATAATCTGTTCTAAAGCCTCCT | | | | |
| LFYChIP-Fd | AGCCAGTATTGCCAACTTTCC | | | | |
| LFYChIP-Rd | TCTTAAGATACATGGCCAACCT | | | | |
| LFYChIP/FAIRE-Fe | tcaccacagtgaaaaccctaatc | | | | |
| LFYChIP/FAIRE-Re | gctgggaaattgacagttgg | | | | |
| **MNase** |  | | | | |
| FIL_MNase_-1F | | TTCTATTGTCAAAGCTTTAAAACT | | | |
| FIL_MNase_-1R | | AATGTGTTTGCAAGTAAGTTAAGAA | | | |
| FIL_MNase_1F | | TCAAGAGCTTCGTCGTTTCTTAAC | | | |
| FIL_MNase_1R | | TAGGCTTTGGGGTTGGGGT | | | |
| FIL_MNase_2F | | CATTACATGTGTCATCACGTGCA | | | |
| FIL_MNase_2R | | AAGGGAAGATTGAGGCATACATTAG | | | |
| FIL_MNase_3F | | CTCGCGAATCTTCTAATGTATGC | | | |
| FIL_MNase_3R | | ATGATCAATTTTGAAATCTTAGGAAG | | | |
| FIL_MNase_4F | | GTACTACAATATCTTCTTCCTAAGA | | | |
| FIL_MNase_4R | | ATGAGATTGGATCAATATAGCGATG | | | |
| FIL_MNase_5F | | AAGTGGTTTGTAGTTTAGTGGTCAT | | | |
| FIL_MNase_5R | | AGCTACTATACAAAACTTTCGACAA | | | |
| FIL_MNase_6F | | TCATATTTTTGTCGAAAGTTTTG | | | |
| FIL_MNase_6R | | GCTATAGGCATTTATGTTTCTATAT | | | |
| FIL_MNase_7F | | TTGATTCTTTAGTTAGAATTTCTTG | | | |
| FIL_MNase_7R | | TGGTCTATACAATTAAAAGCACATG | | | |
| FIL_MNase_8F | | GCTATTTTCATATATTAACATGTGC | | | |
| FIL_MNase_8R | | CTGATCTCAAACAATTACAAACCTA | | | |
| FIL_MNase_9F | | CCAAAATTTAATTGATTGCAAAA | | | |
| FIL_MNase_9R | | GATATTATCGCTTAAAGATGGACCT | | | |
| FIL_MNase_10F | | TGAGATCAGATAGAAAAAGGTCCAT | | | |
| FIL_MNase_10R | | GTGGTCACAAGGAGACAAGACTGAT | | | |
| FIL_MNase_11F | | GCGATAATATCCTCAAAACTA | | | |
| FIL_MNase_11R | | CTATACCGTTGTACCTCTAAGATGA | | | |
| FIL_MNase_12F | | TCAGTCTTGTCTCCTTGTGACC | | | |
| FIL_MNase_12R | | CTAGAAAACGTAGACATTAAGCATC | | | |
| FIL_MNase_13F | | AGGTACAACGGTATAGATGCTTAAT | | | |
| FIL_MNase_13R | | CCTATATTAGAAATTCGGTTGACCA | | | |
| FIL_MNase_14F | | GTAAGGGTGAGACCGCATGG | | | |
| FIL_MNase_14R | | ATTAAATCTTTGTTGTCAACATCCA | | | |
